# Drug-Induced Amyloid-Related Imaging Abnormalities: A Neurovascular Perspective on Risk Assessment

**DOI:** 10.3390/ph19040579

**Published:** 2026-04-03

**Authors:** Marialuisa Zedde, Mattia Losa, Andrea Donniaquio, Ilaria Gandoglia, Massimo Del Sette, Luca Roccatagliata, Fabrizio Piazza, Matteo Pardini, Rosario Pascarella

**Affiliations:** 1 Stroke Unit, Neurology Unit, Azienda Unità Sanitaria Locale-IRCCS di Reggio Emilia, 42123 Reggio Emilia, Italy; 2 “The Inflammatory Cerebral Amyloid Angiopathy and Alzheimer’s Disease Biomarkers” Study Group of the SINDem (Italian Neurological Society for Dementia), 20145 Milan, Italy; 3 Department of Neuroscience, Rehabilitation, Ophthalmology, Genetics, Maternal and Child Health (DINOGMI), University of Genoa, 16132 Genoa, Italy; 4 E.O. Ospedali Galliera, 16128 Genoa, Italy; 5 Neurology Unit, IRCCS Ospedale Policlinico San Martino, 16132 Genoa, Italy; 6 Neuroradiology Unit, Department of Health Sciences, University of Genoa, 16126 Genoa, Italy; 7 CAA and AD Translational Research and Biomarkers Laboratory, School of Medicine and Surgery, University of Milano-Bicocca, 20900 Monza, Italy; 8 Neuroradiology Unit, Ospedale Santa Maria della Misericordia, AULSS5 Polesana, 45100 Rovigo, Italy

**Keywords:** ARIA, amyloid-related imaging abnormalities, cerebral amyloid angiopathy, Alzheimer’s disease, biomarkers, MRI, FLAIR, GRE, SWI, microbleeds, cortical superficial siderosis, apoE, anti-amyloid therapy

## Abstract

Background: Anti-amyloid therapies (AAT) are reshaping the therapeutic landscape of Alzheimer’s disease (AD), yet their implementation remains constrained by the risk of amyloid-related imaging abnormalities (ARIA). Although the ARIA phenomenon is well recognized, most available evidence stems from clinical trial safety reports framed predominantly from a dementia-oriented perspective, with relatively limited integration of vascular neurology principles. Methods: In this narrative review, we examine drug-induced ARIA through a neurovascular lens, highlighting how cerebrovascular comorbidity, particularly cerebral amyloid angiopathy (CAA), influences the risk and severity of ARIA. Results: We critically evaluated how CAA comorbidity has been assessed in randomized controlled trials, focusing on exclusion criteria, imaging thresholds, and the resulting implications for external validity. Finally, we evaluated current approaches to ARIA risk stratification and proposed a more integrative framework that combines vascular imaging markers, APOE ε4 genotype, and key clinical comorbidities. Conclusions: A more tailored patient selection and monitoring strategies may ultimately improve real-world outcomes and optimize resources in the era of AAT.

## 1. Introduction

Anti-amyloid therapies (AAT) are reshaping the clinical and therapeutic landscape of Alzheimer’s Disease (AD) [1]. Several monoclonal antibodies targeting β-amyloid have demonstrated modest but reproducible benefits on cognitive and functional decline in selected patients with early-stage AD [2,3]. Despite these advances, the implementation of AAT remains constrained by a distinct spectrum of treatment-emergent adverse events requiring structured monitoring, collectively termed amyloid-related imaging abnormalities (ARIA) [4]. ARIA includes both exudative changes (ARIA-E), typically manifesting as vasogenic edema or sulcal effusions, and hemorrhagic manifestations (ARIA-H), including cerebral microbleeds (CMB), cortical superficial siderosis (cSS), and lobar intracerebral hemorrhage (ICH).

The pathobiology of ARIA cannot be fully understood without considering the close relationship between AD and Cerebral Amyloid Angiopathy (CAA) [5]. AD and CAA are the two main amyloid-related brain diseases and frequently coexist, particularly in older individuals [6]. Neuropathological series suggest that up to approximately 80% of patients with AD pathology harbor concomitant CAA, indicating that parenchymal plaques and vascular amyloid deposition are not independent processes but rather components of a shared disease spectrum [7]. Most of the evidence informing ARIA risk, monitoring strategies, and management originates from randomized controlled trials (RCTs) in AD, which were designed and interpreted predominantly from a dementia-centered perspective. Trial inclusion and exclusion criteria were primarily constructed to ensure internal validity and safety; however, they often relied on relatively coarse markers of cerebrovascular disease (e.g., microbleed counts or prior hemorrhage) and did not systematically incorporate contemporary diagnostic frameworks for CAA [8,9]. As a result, trial populations may not fully reflect the spectrum of cerebrovascular comorbidity encountered in routine clinical practice, thereby limiting the external validity of ARIA risk estimates. Furthermore, current approaches to ARIA risk stratification in clinical practice remain heterogeneous and only partially grounded in vascular neurology principles [4,10]. Important modifiers—such as systemic comorbidities, vascular risk factors, and frailty- are inconsistently integrated into clinical decision-making and lack standardized implementation across centers.

In this narrative review, we examined drug-induced ARIA through a neurovascular lens. Specifically, we critically appraised how vascular comorbidity was addressed in pivotal clinical trials, highlighting how current trial-derived exclusion criteria may lack accuracy in a more heterogeneous real-world population. We discussed how the clinico-radiological evaluation of vascular co-pathology could be refined by integrating contemporary diagnostic frameworks, proposing a more tailored and biology-driven ARIA risk assessment process. Reassessing the conceptual and practical foundations of ARIA risk evaluation is essential, given its substantial impact on patient eligibility for AAT.

## 2. Methods

Given the evolving nature of the field and the conceptual gaps in current trial data, this work is structured as a narrative review. A comprehensive literature search was conducted on 1 February 2026, using the PubMed database and a manual review of reference lists from pertinent articles. The search strategy employed the search terms: ((“Amyloid-Related Imaging Abnormalities”[tiab] OR ARIA[tiab] OR “ARIA-E”[tiab] OR “ARIA-H”[tiab] OR (“amyloid”[tiab] AND “neuroimaging”[tiab])) AND (“Cerebral Amyloid Angiopathy”[majr] OR (“Anti-Amyloid Treatment”[tiab] OR “Anti-Amyloid Therapy”[tiab] OR “Monoclonal Antibodies”[majr] OR lecanemab[tiab] OR donanemab[tiab])). Only original research papers published in English were reviewed. The final reference list was selected based on relevance to the scope of this review.

## 3. Drug-Induced and Spontaneous ARIA: Differences and Similarities

The term ARIA was originally introduced in the context of immunotherapy trials for AD to describe MRI changes emerging under treatment [11]. Subsequent RCTs of monoclonal antibodies, including aducanumab, donanemab, and lecanemab, confirmed that these imaging findings represent a class effect of anti-amyloid therapies (AAT) and formalized the radiological distinction between ARIA-E (edema/effusion) and ARIA-H (hemorrhage) [2,3].

On brain MRI, AAT-associated ARIA-E and ARIA-H closely resemble the spontaneous inflammatory manifestations of CAA, collectively termed CAA-related inflammation (CAA-ri) [12]. Clinically, most cases of drug-induced ARIAs are asymptomatic or only mildly symptomatic and are detected through protocol-mandated MRI surveillance rather than by the onset of new neurological symptoms [13]. When symptoms occur, they are typically mild and often resolve following dose interruption or reduction and, in selected cases, with corticosteroid therapy. In contrast, detection of spontaneous ARIA in the context of CAA-ri is typically symptom-driven. Patients commonly present with subacute encephalopathy, cognitive or behavioral changes, focal neurological deficits, or seizures [14]. Nevertheless, large case series indicate that many patients present with a single, and sometimes mild, symptom [12].

Despite differences in triggering mechanisms and clinical context, AAT-associated ARIA and CAA-ri share a nearly identical radiological profile and a closely related biological substrate [15]. In both settings, a convergence of factors, including abundant vascular Aβ deposition (CAA), mobilization or redistribution of amyloid (mediated either by exogenous monoclonal antibodies or endogenous anti-Aβ autoantibodies), activation of microglia and astrocytes, and disruption of the blood–brain barrier (BBB)—ultimately leads to vasogenic edema and an increased propensity for hemorrhage [16,17,18,19]. Pathological specimens from AAT-treated patients with ARIA and from patients with CAA-ri show a similar inflammatory signature, characterized by perivascular lymphocytic infiltrates, CD68-positive macrophages, activated microglia and astrocytes, fibrinoid necrosis, and evidence of BBB breakdown, further supporting a shared pathophysiological framework, defined as the ARIA paradox model [4,20,21,22,23,24].

This overlap has important implications. Accumulating evidence indicates that ARIA and CAA-ri are not distinct conditions, but rather different clinical manifestations of a shared Aβ-driven, immune-mediated vasculopathy. ARIA represents the neuroradiological expression of this process in the context of therapeutic amyloid mobilization, whereas CAA-ri reflects its spontaneous, clinically overt form. In this framework, anti-amyloid therapy does not create a new pathological entity; instead, it precipitates or amplifies an inflammatory response within an already amyloid-laden and structurally vulnerable cerebral vasculature [4].

## 4. Eligibility Criteria in AAT Clinical Trials from the Neurovascular Perspective

The principal exclusion criteria of neurovascular relevance in the lecanemab and donanemab randomized controlled trials [2,3] are detailed in Table 1.

As evident from the reported tables, the radiological selection criteria applied in RCTs [2,3] were intended to exclude patients with overt vascular encephalopathy (including radiological manifestations suggestive of CAA), with two main purposes:

(i) First, to exclude patients in whom a vascular component may have played a role in the etiology of the cognitive impairment;
(ii) Second, since ARIA-E and brain hemorrhage are the most feared complications of AAT, RCTs attempted to exclude patients with plausible ARIA risk factors (overt cerebral SVD) or inadequately compensated hemorrhagic risk factors (e.g., hemostasis issues, uncontrolled arterial hypertension).

Another major topic regarding AAT is antithrombotic therapy. This has been discussed in more depth in previous reviews and recommendations, to which we refer [10,25,26,27]. To sum up, even if RCTs did not explicitly state exclusion criteria for patients on antithrombotic therapy, both lecanemab and donanemab recommendations from the regulatory agencies stated that: (1) lecanemab and donanemab are not recommended in patients on anticoagulant therapy, while antiplatelet therapies are permitted; (2) if anticoagulation needs to be commenced during therapy (for example, incident arterial thromboses, acute pulmonary embolism, or other life-threatening indications), the AAT should be paused. AAT can be reinstated if anticoagulation is no longer medically indicated; (3) use of thrombolytic agents should be avoided except for immediately life-threatening indications with no alternative management (e.g., pulmonary embolism with hemodynamic compromise) when the benefits could outweigh the risks. Patients on anticoagulant therapy were rarely enrolled in RCTs, so the safety profile of either drug in this subgroup of patients is unknown.

Applying the selection criteria from RCTs or appropriate use recommendations (AUR) [28,29,30] retrospectively, the eligibility for AAT among a population of MCI or mild dementia due to AD ranged from 8% to 24% [31,32,33]. Although AAT eligibility criteria were designed to enhance safety through stringent patient selection, the extent to which these criteria specifically capture concomitant CAA as a main risk factor for ARIA remains uncertain.

## 5. Limitations of RCTs’ Selection Criteria from a Neurovascular Perspective

By examining the different SVD-related markers listed in the exclusion criteria for AAT, some considerations relevant from a neurovascular viewpoint can be discussed. Although the primary objective of imaging-based exclusion criteria is to identify patients with CAA, this is not explicitly mentioned in RCTs. Furthermore, the MRI-related exclusion criteria are not coincident with the diagnostic criteria for CAA [8,34,35,36,37].

The CMB location is important for both diagnostic and prognostic purposes [38]. Indeed, from a histopathological standpoint, CMBs themselves are characterized by heterogeneous pathologic substrates [39,40], with neuroradiological investigation capable of suggesting the underlying microangiopathic process based on the location of CMBs. In fact, the spatial distribution of CMBs tends to parallel the preferential vascular involvement of CAA, mainly affecting cortical and leptomeningeal vessels, or of arteriolosclerosis, which mainly involves deep brain regions. Thus, the anatomical location is considered a useful MRI biomarker to differentiate the two major forms of sporadic SVD [41]. Given the high positive predictive value for CAA, a hospital-based setting (specificity 90%), strictly lobar distribution of CMBs has been included in the pathology-proven diagnostic criteria for CAA [8].

Nevertheless, in the absence of an appropriate clinical context (i.e., ICH, convexity subarachnoid hemorrhage, transient focal neurological episodes, and cognitive impairment), the overall specificity is low, as lobar CMBs can also be found in the absence of CAA [41]. Moreover, even in a strictly lobar location, there is a difference between a cortical and a subcortical location of CMBs, the former being more strongly associated with the diagnostic hypothesis of CAA [42] and the latter with arteriolosclerosis [43].

The concomitant deep and lobar location of CMBs, the so-called mixed SVD, has been associated with vascular risk factors, given the predominant arteriolosclerosis pathophysiology in most (but not all) the cases [37,44,45]. The cerebellum may be affected by both types of SVD [46,47].

Despite all these considerations, no study has explored to date the comparative predictive value of deep versus lobar CMBs as a risk factor for ARIA.

CMBs have a relatively high prevalence in several subgroups. Considering a population setting, the reported prevalence of CMBs in elderly populations ranges from 5% to 35% [48,49,50,51] and cumulative incidences of new CMBs of approximately 7–10% over a period of 3–4 years [50,51]. The reported location of CMBs was predominantly lobar, ranging from 48% in the Northern Manhattan Study to 70% in the Age, Gene/Environment Susceptibility–Reykjavik Study [48,49]. Deep and mixed location CMBs (i.e., lobar and deep) ranged from 32% in the Rotterdam Scan Study [50,51] to 52% in the Northern Manhattan Study [49]. In stroke-free individuals, the overall presence of CMBs is associated with a 5.5-fold and 2-fold increased risk of first spontaneous ICH and first ischemic stroke (IS), respectively [52]. Interestingly, the location of CMBs is important: while lobar CMBs are associated with a fivefold increased risk of sICH, they do not seem to influence the IS risk. In contrast, deep CMBs are associated with a 6-fold increased risk of sICH and a 2.5-fold increased risk of IS [51].

About the number of CMBs, the threshold set in the AAT eligibility criteria (i.e., 4) does not correspond to a minimum number of CMBs necessary to fulfill the Boston criteria v2.0 [8]. Given this, the threshold of four, defined by the trials’ exclusion criteria, is somewhat arbitrary, although likely influenced by previous studies about the risk of ICH during oral anticoagulant therapy and also supported by recent exploratory analysis on donanemab data [13,53]. Another variable is strictly technological; in fact, there is considerable variability in the sensitivity of CMB identification, which increases with the scanners’ magnetic field strength, and is also influenced by the sequence type (GRE vs. SWI) [54]. Some consensus recommendations have emerged since the publication of the RCTs to limit variability in clinical practice [55,56,57,58,59].

Cortical superficial siderosis is the most specific radiological feature of CAA, in view of its strong correlation with CAA-related pathological changes [8], and it has been clearly associated with hemorrhagic risk, in particular when disseminated [60]. Even if cSS is a much more specific marker for CAA than CMBs, the presence of cSS does not automatically mean CAA and cSS have a multitude of differential diagnoses, similarly to convex subarachnoid hemorrhage (cSAH) [8].

Also, non-hemorrhagic SVD-related markers are considered in the AAT neuroradiological exclusion criteria, in particular, the presence of severe WMHs and lacunar infarcts. About WMH, severe small vessel disease was translated into a Fazekas score of 3 in the post-marketing operational instructions, to ensure comparability, but the anatomical distribution of WMH (e.g., posterior-predominant WMH) has never been evaluated as an ARIA predictor [61,62]. The numerical threshold for lacunes, as for CMB, was arbitrary, and the potentially different effects of lacune sites are not defined. There is no emphasis on excluding infarcts in strategic territories [63], which can have significant consequences due to lesions at nodal points (e.g., thalamic location [64,65], claustrum [66]) [67,68,69].

**Figure 1:**
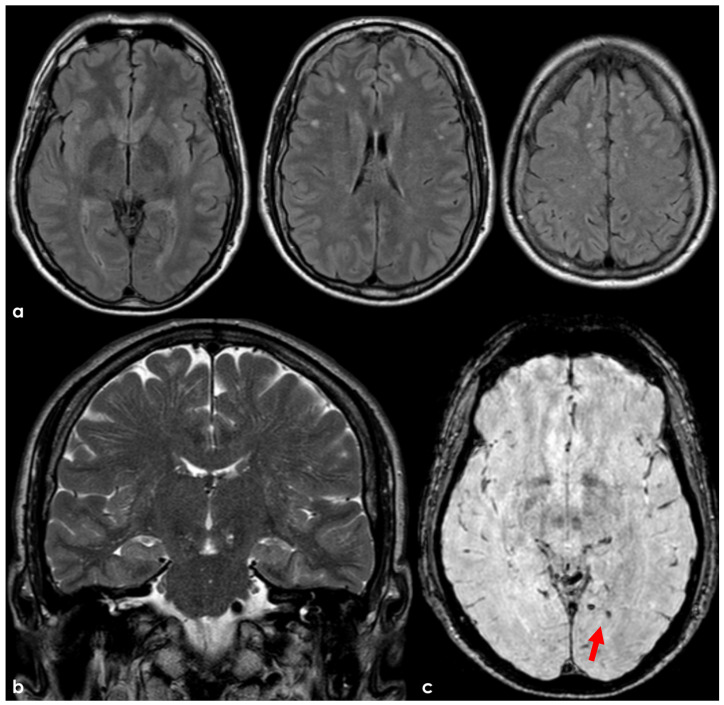
MRI of a patient fulfilling the diagnostic criteria for “possible CAA” and no restrictions for enrollment in Lecanemab and Donanemab treatment. Panel (**a**): axial FLAIR MRI showing a few punctate WMHs with a prevalent subcortical location, which resembles a WMH-MS. Panel (**b**): coronal T2W sequence with slightly enlarged perivascular spaces in the centrum semiovale. Panel (**c**): axial SWI with a single left temporal microbleed (red arrow).

**Figure 2:**
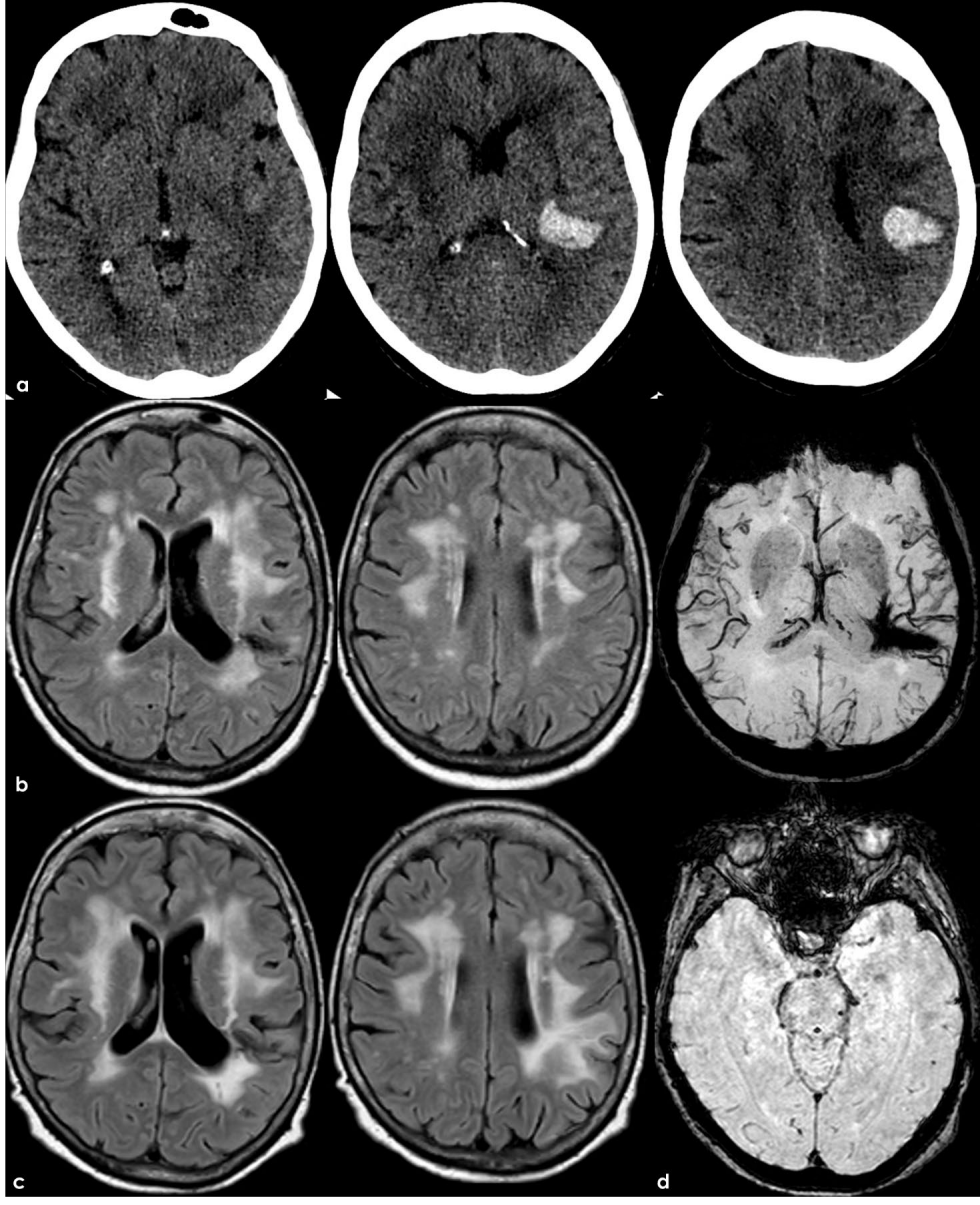
Non-contrast CT (NCCT) and MRI findings in a patient with CAA-related intracerebral hemorrhage (ICH), and CAA-related inflammation (CAA-ri). Panel (**a**): Axial NCCT images (displayed from inferior to superior, left to right) demonstrate confluent symmetrical white matter hypodensities consistent with small vessel disease and a hyperdense acute intracerebral hemorrhage located at the left temporo-parieto-frontal junction. Panel (**b**): Axial FLAIR MRI obtained three months later confirms confluent, symmetrical supratentorial white matter hyperintensities (WMHs). Panel (**c**): Axial FLAIR MRI performed two years later reveals a left posterior frontal white matter hyperintensity with mild mass effect, consistent with spontaneous ARIA-E. The MRI was acquired following new-onset refractory status epilepticus (NORSE), supporting the clinical suspicion of CAA-ri. Panel (**d**): Axial susceptibility-weighted imaging (SWI), reconstructed using a minimum intensity projection (minIP) protocol, shows the residual hypointense cavity from the prior ICH and a single left temporal cerebral microbleed.

The Boston criteria v2.0 [8] introduced in 2022 the WMH in the form of a subcortical multispot pattern (WMH-MS), and the enlarged perivascular spaces of the centrum semiovale (CSO-EPVS) among the non-hemorrhagic markers supportive of CAA diagnosis. Even if these MRI markers present a good predictive value for an underlying CAA in the correct clinical context [70], they are not currently mentioned in the AAT recommendation. In fact, a patient with a WMH-MS usually qualifies for an associated deep white matter Fazekas scale of 1, allowing in most of the cases to be recruited for AAT. At the same time, some studies have shown that the latest Boston criteria v2.0 probably present a suboptimal sensitivity and specificity compared to pathological diagnosis in the context of a non-hemorrhagic presentation [71,72]. Still, some patients with a formal “possible” or even “probable CAA” diagnosis (Figure 1) would be currently eligible for AAT, and no data have been published about these diagnostic categories and ARIA risk implications. Conversely, patients with overt CAA (Figure 2) are clearly ineligible for AAT, demonstrating the wide range of severity encompassed by the probable CAA category. Interestingly, most of the factors affecting the progression from the status of Figure 1 to that of Figure 2 are not well known in individual cases.

Guidelines of regulatory agencies and AUR [25,26,29,30] explicitly refer to other conditions that may increase the hemorrhagic risk as elements to be carefully evaluated before determining the patient’s eligibility for treatment, explicitly mentioning other lesions (such as aneurysms, vascular malformations) that could potentially increase the risk of ICH. Both aneurysms and cavernous angiomas are vascular malformations that are far from rare in the adult population and represent one of the most common incidental findings in neuroimaging examinations conducted for other reasons. The prevalence of unruptured intracranial aneurysms in the general population is between 2.3% and 10.9% [73], with variable SAH risk depending on location and size [74,75], as demonstrated by the International Study of Unruptured Intracranial Aneurysms (ISUIA) [75], which prospectively evaluated 1692 patients with 2686 unruptured and untreated aneurysms in the USA, Canada, and Europe, and the Unruptured Cerebral Aneurysms Study (UCAS) [76], a Japanese cohort of 6697 aneurysms in 5720 patients. Both studies established a clear relationship between the location and size of the aneurysm and the risk of rupture. The PHASES score, developed from a pooled analysis of six prospective cohort studies, incorporated age, hypertension, maximum aneurysm diameter, previous SAH history, and aneurysm location as key predictors of rupture risk, providing a useful tool for individualized treatment strategies [77]. On the other hand, cavernous angiomas are mostly congenital vascular malformations, with a relatively low risk of bleeding if detected as incidental findings (under 1% annually). The lifelong bleeding risk is significantly higher after a prior hemorrhage (4–25% yearly), with increased risks for deeper locations (brainstem), females, younger age, and certain genetic forms (CCM3) [78,79,80]. Although it is not stated as an absolute exclusion criterion for patient enrollment in the RCTs, there is insufficient information on the safety profile of lecanemab and donanemab in this subgroup of patients. Recently, a single case report was published regarding a patient treated with lecanemab despite having an incidental finding of a cavernous angioma [81], which exhibited an asymptomatic increase in size with subacute blood products without additional new ARIA, resulting in treatment discontinuation. Further studies should evaluate safety in patients with vascular malformation.

Some recently published studies used retrospective cohorts to apply the selection criteria of AAT RCTs [2,3] to the Memory Clinics patients, aiming to estimate the proportion of eligible patients in a real-world setting. Applying the eligibility criteria of the CLARITY-AD trial [2] to participants with early AD from the Mayo Clinic Study of Aging (MCSA) [31], only 8% of the initial cohort. The most common reasons for exclusion were chronic diseases and neuroimaging findings, using T2* GRE sequences on 3 T scanners for quantifying CMBs [82]. In a similar study on a European cohort [32], the proportion of patients eligible for AAT varied from 8% (6.2–9.9) for lecanemab to 15% (12.4–17.5) for donanemab. Furthermore, the proportion of patients not eligible based solely on neuroradiological criteria was about 1/3, mainly conditioned by the presence of SVD, using T2*-GRE sequence on 1.5-T MRI scanners [32,83]. Additionally, in an Asian cohort of 1005 A+ subjects [33], about 1/3 presented neuroradiological exclusion criteria (primarily SVD).

## 6. ARIA Risk Assessment: Present and Future

### 6.1. Current Approach to Pre-Treatment ARIA Risk Assessment

In clinical practice, as stated above, patient evaluation for AAT eligibility usually relies on strict criteria largely inherited from pivotal RCTs and included in regulatory guidance [29,30]. After a diagnosis of MCI or mild dementia due to AD based on current diagnostic criteria [84,85], the patient is usually referred for consideration of AAT. Baseline work-up generally follows the standard algorithm derived from RCTs, which includes:

(i) A clinical assessment to exclude major comorbidities (e.g., recent ischemic stroke or TIA, active cancer, systemic autoimmune disease, anticoagulant therapy, uncontrolled arterial hypertension);
(ii) APOE genotyping (which is recommended by the FDA but mandatory for EMA)
(iii) A pre-treatment MRI to evaluate hemorrhagic and ischemic features.

According to EMA, if the pre-treatment MRI shows more than four CMB, any cSS or prior ICH (>1 cm), more than 2 lacunes, or severe WMH (namely, deep with matter Fazekas scale greater than 2 [86]), the patient is typically excluded from lecanemab or donanemab treatment. If not, the patient is considered eligible and, after a discussion with the patient and the caregiver about risks/benefits balance, based on genotype and radiological profile, AAT can be initiated. AATs are usually administered under the supervision of a multidisciplinary team trained in ARIA management.

As mentioned before, these recommendations focus primarily on a limited set of clinical and imaging-based exclusion criteria and, in some countries, on homozygous APOE e4 genotype. While these criteria ensure safety in RCT settings, they have possible improvable accuracy in identifying real predictive factors that shape vascular vulnerability and, by extension, ARIA risk.

### 6.2. ARIA Risk Assessment: Possible Implementations from a Vascular Perspective

The current screening approach is pragmatic, but it is fundamentally trial-driven rather than biology-driven, and it assumes that safety criteria designed for highly selected RCT populations can be directly applied to the heterogeneous real-world setting. To sum up, Table 2 shows the possible limitations of the current framework:

The current approach, based on a checklist of single items, may miss the substantial neurovascular heterogeneity among patients, particularly the large proportion of older adults with varying degrees of non-overt CAA. As mentioned, several possible markers of ARIA risk susceptibility, such as the presence and extent of CSO-EPVS and white-matter changes consistent with CAA (WMH-MS or posterior-predominant WMH), are unexplored as risk factors so far [13]. Additionally, considering only single factors (e.g., radiological features, APOE genotype, medical comorbidities) may under- or overestimate the global single-patient risk of ARIA, not accounting for interactions among imaging, biological, and genetic elements, and avoiding a real patient-specific ARIA risk stratification.

Moreover, a high vascular risk profile is not a formal contraindication to AAT, although such patients are treated with AAT with greater hesitations, due to possible incidental indications to antithrombotics [88]. At the same time, poorly controlled hypertension is a significant modifiable risk factor for ARIA occurrence [13]. In view of this, the AAT journey should also encompass a pre-treatment comprehensive evaluation and optimization of vascular risk factors to minimize the probability of cardiovascular disease occurrence.

Taken together, these limitations result in a possibly incomplete approach to ARIA risk assessment that does not fully capture the neurovascular mechanisms driving biological susceptibility to ARIA. In this view, a more tailored ARIA risk assessment may be able to drive personalized pre-treatment counseling on risk/benefit discussion and also drive the MRI surveillance intensity. This second point is particularly relevant given the possible reduction in logistics and cost implications related to AAT.

### 6.3. Future Directions

**Figure 3:**
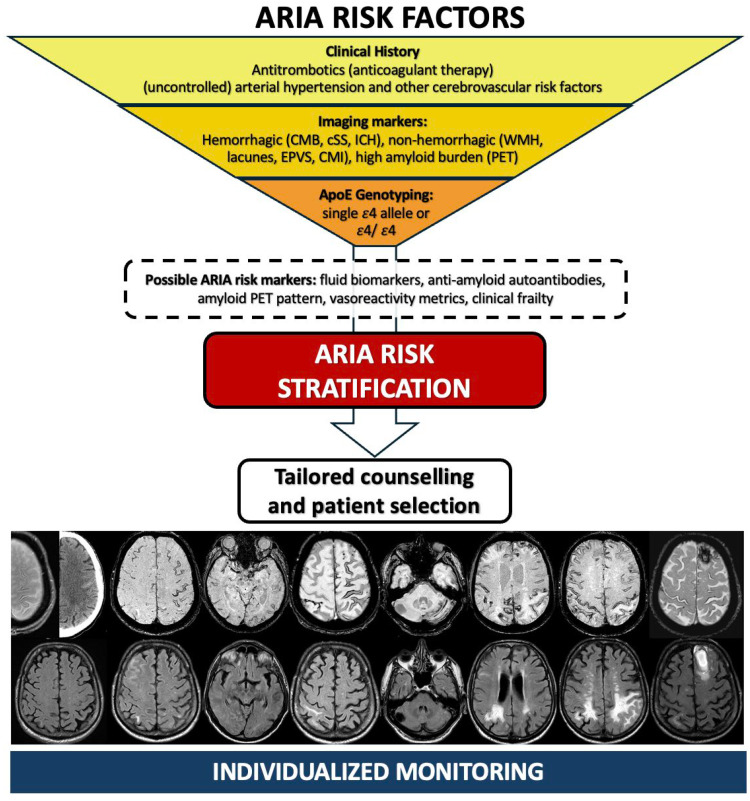
ARIA risk stratification: main elements considered and future perspectives. Legend: ARIA = Amyloid-Related Imaging Abnormalities; CMB = Cerebral Microbleeds; CMI = Cerebral Microinfarcts; cSS = cortical superficial siderosis; EPVS = Enlarged Perivascular Spaces; ICH = Intracerebral Hemorrhages; PET = Positron Emission Tomography; WMH = White Matter Hyperintensities.

Classical hemorrhagic and non-hemorrhagic MRI markers are markers of advanced CAA, unable to detect preclinical CAA (MRI-negative CAA), and poorly sensitive for mild CAA [89]. Several unexplored markers may further improve risk assessment, such as the applications of advanced imaging sequences, including dynamic contrast-enhanced MRI for BBB permeability [90], occipital-predominant distribution of tracer uptake detected using amyloid PET [91], fluid biomarker (e.g., microglial activity evaluated by sTREM2 or YKL-40 levels [92,93], CAA-related CSF biomarker pattern [90,94]), or specific CAA-ri fluid biomarkers [4,16]. In the future, a more comprehensive and clinically meaningful strategy should integrate insights from CAA biology, neuroimaging, and genetics toward an integrated ARIA risk assessment model (Figure 3).

## 7. Conclusions

This review focused on ARIA and drug-induced risk assessment from a neurovascular perspective. We proposed to shift from rigid, trial-derived checklists for AAT eligibility toward an integrated, biology-driven framework that captures a dynamic continuum of neurovascular risk. By synthesizing imaging markers, genetics, and clinical comorbidities, we advocate for a personalized approach to patient selection for AAT. Reframing the ARIA risk assessment through a neurovascular lens and embedding CAA-focused markers within ARIA risk-stratification frameworks may enable safer and more effective implementation of AAT. Rather than treating ARIA as an adverse event, it should be interpreted as an iatrogenically induced manifestation of CAA-ri, presumably driven by the background pre-existing vascular amyloid burden, even when this is not overtly recognized on baseline imaging. Combining cognitive and neurovascular elements would allow us to move toward a tiered risk model, supporting individualized decisions regarding patient selection, treatment decision making, and MRI safety monitoring plans.

## Figures and Tables

**Table 1:** Main neurovascular-related exclusion criteria applied in the lecanemab and donanemab RCTs [2,3].

Lecanemab [2]	Donanemab [3]
**Exclusion criteria (medical history)**	
- History of TIA, stroke, or seizures within 12 months of screening - Participants with a bleeding disorder that is not adequately controlled (including a platelet count < 50,000 or INR > 1.5 for participants who are not on anticoagulant treatment, e.g., warfarin) - Any other medical conditions (e.g., cardiac, respiratory, gastrointestinal, renal disease) which are not stably and adequately controlled, or which could affect the participant’s safety or interfere with the study assessments - BMI ≤ 17 and ≥35 at screening	- Current serious or unstable illnesses, including cardiovascular, hepatic, renal, gastroenterologic, respiratory, endocrinologic, neurologic (other than AD), immunologic, or hematologic disease, and other conditions that, in the investigator’s opinion, could interfere with the analyses in the study; - Life expectancy of <24 months.
**Exclusion criteria (MRI features at screening)**	
- More than 4 microhemorrhages (defined as 10 mm or less at the greatest diameter); - a single macrohemorrhage > 10 mm at greatest diameter; - an area of superficial siderosis; - evidence of vasogenic edema; - multiple lacunar infarcts or stroke involving a major vascular territory; - severe small vessel; - other major intracranial pathology	- More than 4 cerebral micro-hemorrhages - more than 1 area of superficial siderosis - any intracerebral hemorrhage greater than 1 cm - severe white matter disease - any amyloid-related imaging abnormalities of edema/effusion (ARIA-E)
Participants who are on anticoagulant therapy should have their anticoagulant status optimized and be on a stable dose for 4 weeks before screening	

**Table 2:** Limitations of the current framework for estimating the risk of ARIA.

Issues	Limitations
CMB number	Crude reliance on CMB counts, with a threshold of “>4 CMBs”, was decided discretionally and not based on biological evidence. While the number of CMB is a risk factor for ARIA, there are no data on the application of different thresholds as selection criteria for AAT.
CMB location	They do not account for the anatomical pattern of distribution, which suggests the main etiological driver of the hemorrhagic markers
Natural history	The dynamic changes (historical progression) of the hemorrhagic markers are not considered, which carry stronger implications for CAA evidence [87];
CAA diagnostic criteria	There is limited integration in this setting of CAA-specific non-hemorrhagic imaging markers and diagnostic categories, according to Boston Criteria v2.0 [8].

## Data Availability

No new data were created or analyzed in this study. Data sharing is not applicable to this article.
